# Supplementary material for: Combination of 3D and 2D Small and Wide Angle X‐Ray Scattering Imaging Reveals Diminished Bone Quality in the Superior Human Femoral Neck Cortex
Source: Adv Mater. 2026 Jul 9;38(45):e73848. doi: 10.1002/adma.73848 (PMC13471814; doi:10.1002/adma.73848)
Supplement: Supplementary file 1 [file ADMA-38-e73848-s001.pdf]

Supporting Information

Combination of 3D and 2D small and wide angle x-ray scattering imaging reveals diminished bone quality in the superior human femoral neck cortex

*Torne Tänzer Tatiana Kochetkova Arthur Baroni Mathieu Simon Santiago F. Bordin Manuel Guizar-Sicairos Philippe Zysset Marianne Liebi**

((Please insert your Supporting Information text/figures here. Please note: Supporting Display items, should be referred to as Figure S1, Equation S2, etc., in the main text…)

Table S1: Table summarizing the meansured and derived parameters from SAXS/WAXS and their relationship to bone structure. The parameters marked in bold are employed in the statistical analysis of the whole dataset.

Parameter	Scattering feature	Bone Tissue Feature
**Mer. Collagen Mean Intensity**	Overall intensity from MCF D-spacing scattering on detector (signal 1 in figure 1)	Related to quantity and distribution of MCF in beam path
**Mer. Collagen Degree of Orientation**	Anisotropy of MCF D-spacing scattering on detector (signal 1 in figure 1)	Related to distribution of MCF in beam path
Mer. Collagen Fractional Anisotropy	Anisotropy of MCF D-spacing scattering in voxel after SASTT reconstruction (signal 1 in figure 1)	Anisotropy of MCF distribution in voxel
**Equ. Collagen Mean Intensity**	Overall intensity from MCF fibre scattering on detector (signal 2 in figure 1)	Related to quantity, distribution and packing of MCF in beam path
**Equ. Collagen Degree of Orientation**	Anisotropy of MCF fibre scattering on detector (signal 2 in figure 1)	Related to distribution of MCF in beam path
**Mineral Mean Intensity**	Overall intensity from mineral scattering on detector (signal 3 in figure 1)	Related to quantity and distribution of mineral particles in beam path
**Mineral Degree of Orientation**	Anisotropy of mineral scattering on detector (signal 3 in figure 1)	Related to distribution of mineral particles in beam path
Mineral Fractional Anisotropy	Anisotropy of mineral scattering in voxel after SASTT reconstruction (signal 3 in figure 1)	Anisotropy of mineral particle scattering distribution in voxel
**HA (002) Mean Intensity**	Overall intensity from HA (002) diffraction on detector (signal 4 in figure 1)	Related to quantity and distribution of hydroxyapatite crystals in beam path
**HA (002) Degree of Orientation**	Anisotropy from HA (002) diffraction on detector (signal 4 in figure 1)	Related to distribution of hydroxyapatite crystals in beam path
MCF In-plane Orientation	Orientation of MCF D-spacing scattering on detector (signal 1 in figure 1)	In-plane orientation (in transverse plane of femoral neck) of MCF
**MCF Out-of-plane Angle**	Estimated out-of-plane angle of MCF D-spacing scattering (signal 1 in figure 1)	Out-of-plane angle (with respect to the transverse plane of the femoral neck) of MCF
**T-parameter**	Thickness of mineral particles, assuming plate like mineral scattering (signal 3 in figure 1)	Average thickness of mineral particles in beam path (2D) or voxel (3D)
**Mineral Short-range Ordering Parameter $2\pi/\alpha$**	Ordering of mineral particles, assuming plate like mineral scattering (signal 3 in figure 1)	Correlates with the degree of ordering between successive platelets along the beam path (2D) or within a voxel (3D)
**Inter-platelet Distance $T2\pi/\beta$**	Average distance between mineral particles, assuming plate like mineral scattering (signal 3 in figure 1)	Average distance between successive mineral particles along the beam path (2D) or within a voxel (3D)
**Mer. Collagen D-spacing**	Position of MCF D-spacing scattering peak on detector	Collagen D-spacing
**Equ. Collagen D-spacing**	Position of MCF fibre scattering peak on detector	Diameter of collagen fibres
**Collagen ratio (Mer. vs Equ.)**	Ratio of overall intensities from MCF D-spacing and fibre scattering	Related to stiffness of MCF in beam path (2D) or voxel (3D)
**Crystal Size**	Peak broadening of HA (002) diffraction on detector	Estimation of size of hydroxyapatite crystal domains in beam path
**HA (002) D-spacing**	Position of HA (002) diffraction peak on detector	Lattice plane spacing of the HA (002) planes in the beam path

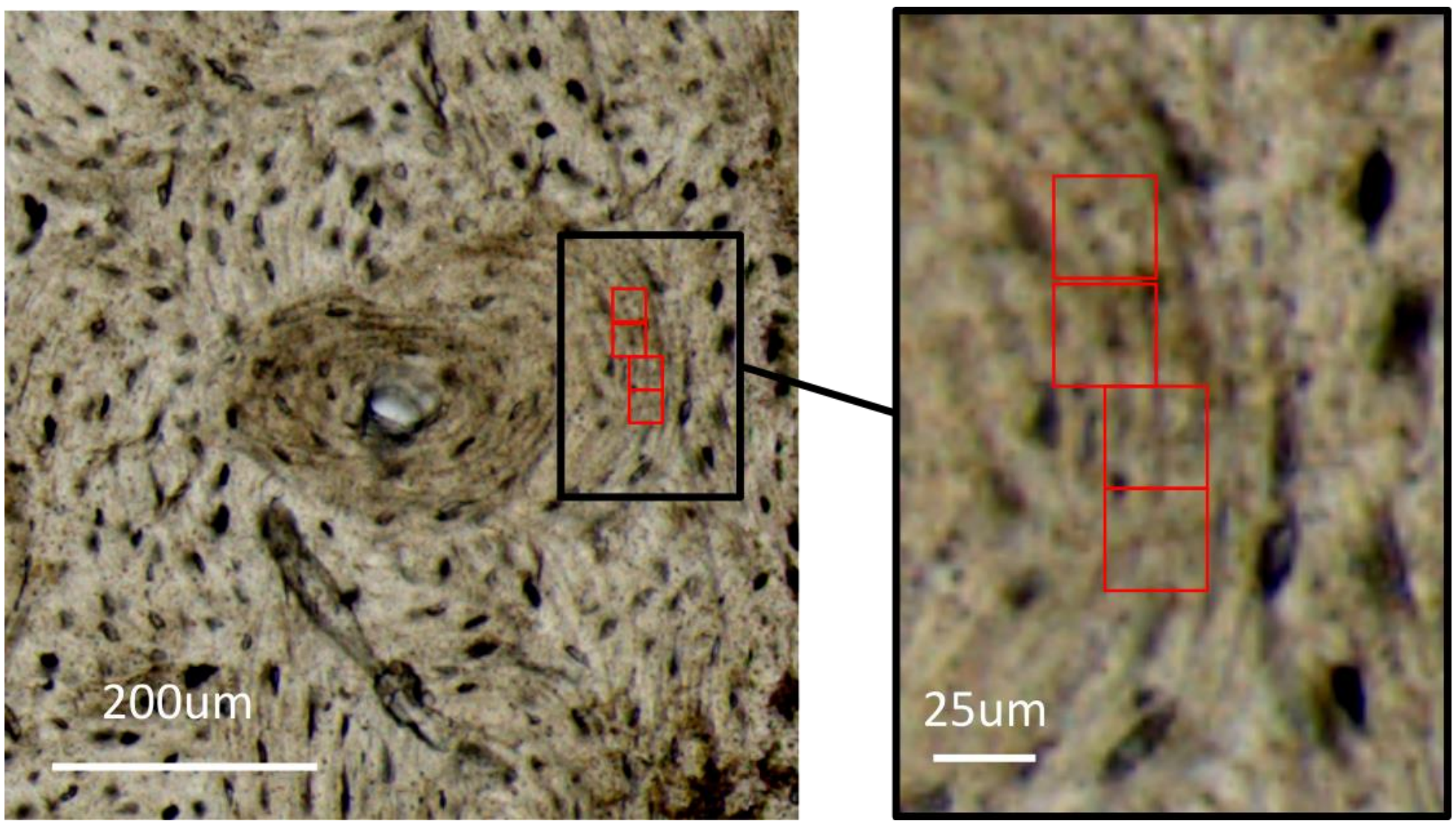

Figure S1: Representation of beamsize/voxel size of 25μm² compared to the bone microstructure. The voxel corresponds to a size in which lamellae are predominantly aligned in parallel, exhibiting minimal curvature over this length scale, leading to non-axially symmetric fibre distributions in the transverse plane.

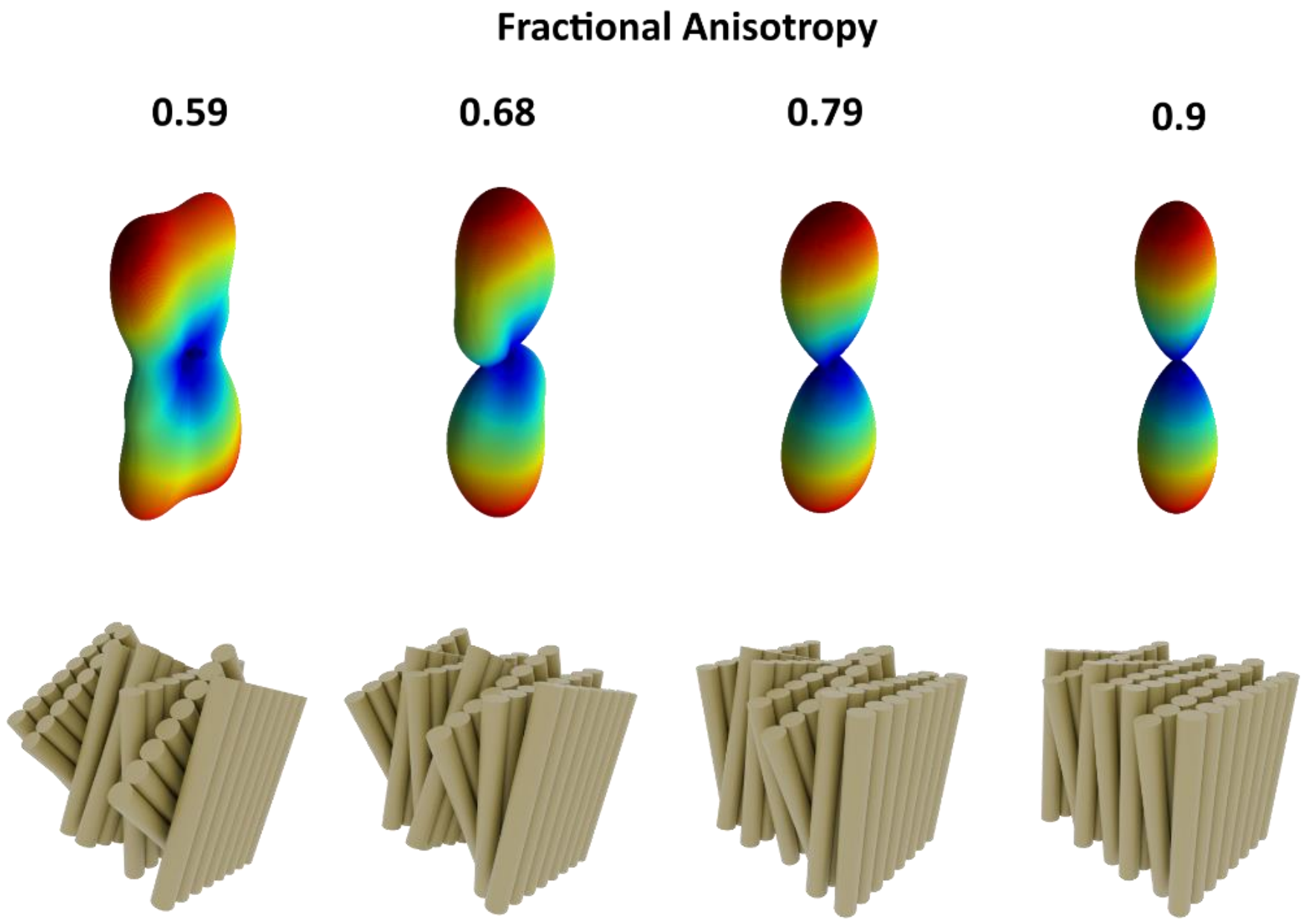

Figure S2: Representative meridional collagen RSM's of different 3D Fractional Anisotropies extracted from the inferior side of sample pair 2 in relation to the possible underlying lamellar structure

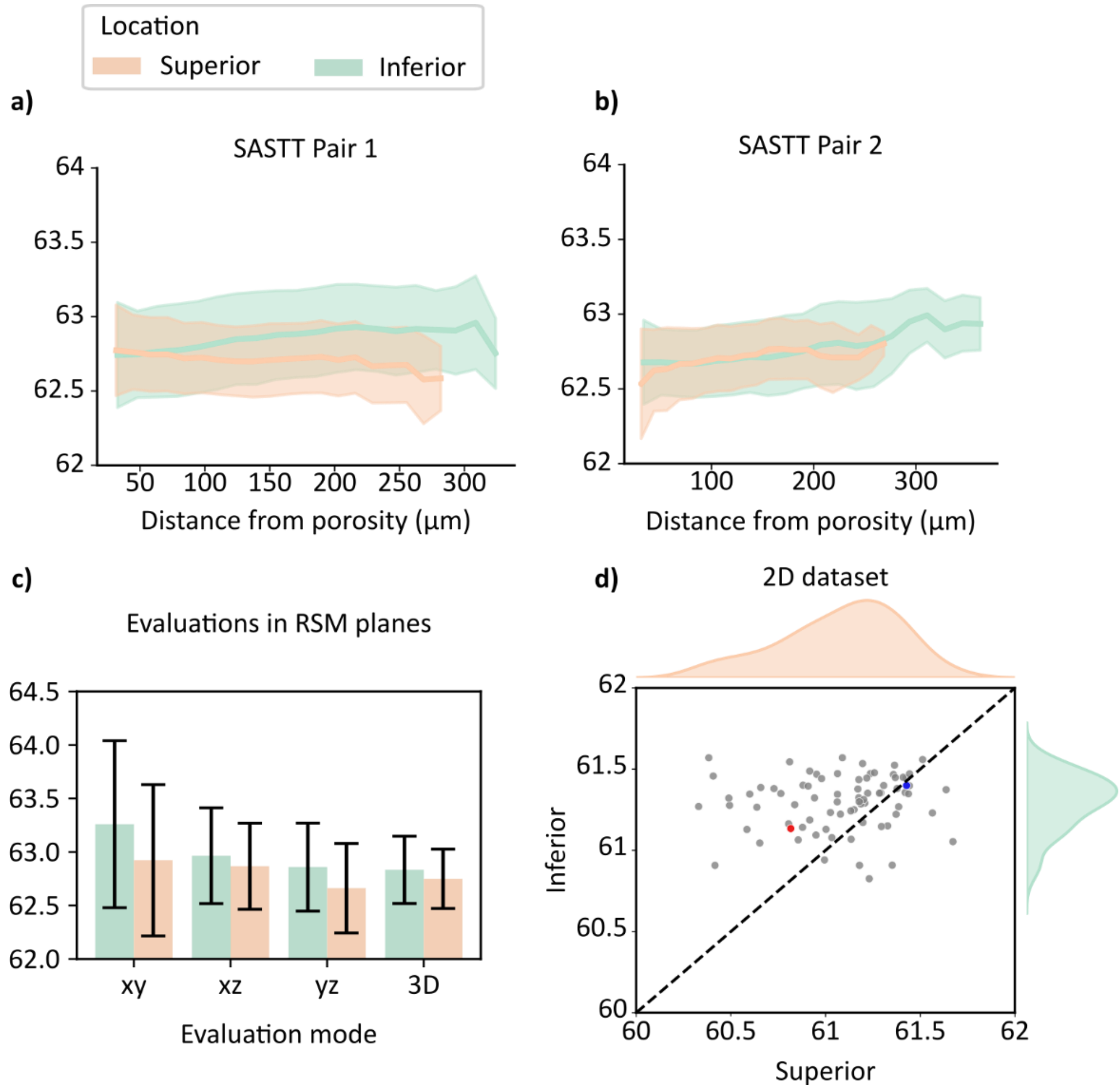

Figure S3: (a,b) Collagen D-spacing (nm) in the SASTT sample pairs, in function of distance from porosity. In pair 1, the collagen D-spacing is slightly lower on the superior side, while they are similar in pair 2. (c) Evaluation of collagen D-spacing in 3 orthogonal planes oriented w.r.t the bone axis z. No significant differences were noted.(d) Scatter plot of the mean D-spacing of all femoral necks (x-axis: superior, y-axis: inferior). There is a significant but very small (0.3%) decrease in the average on the superior side. The blue and red points indicate the sample pairs from figure 4.

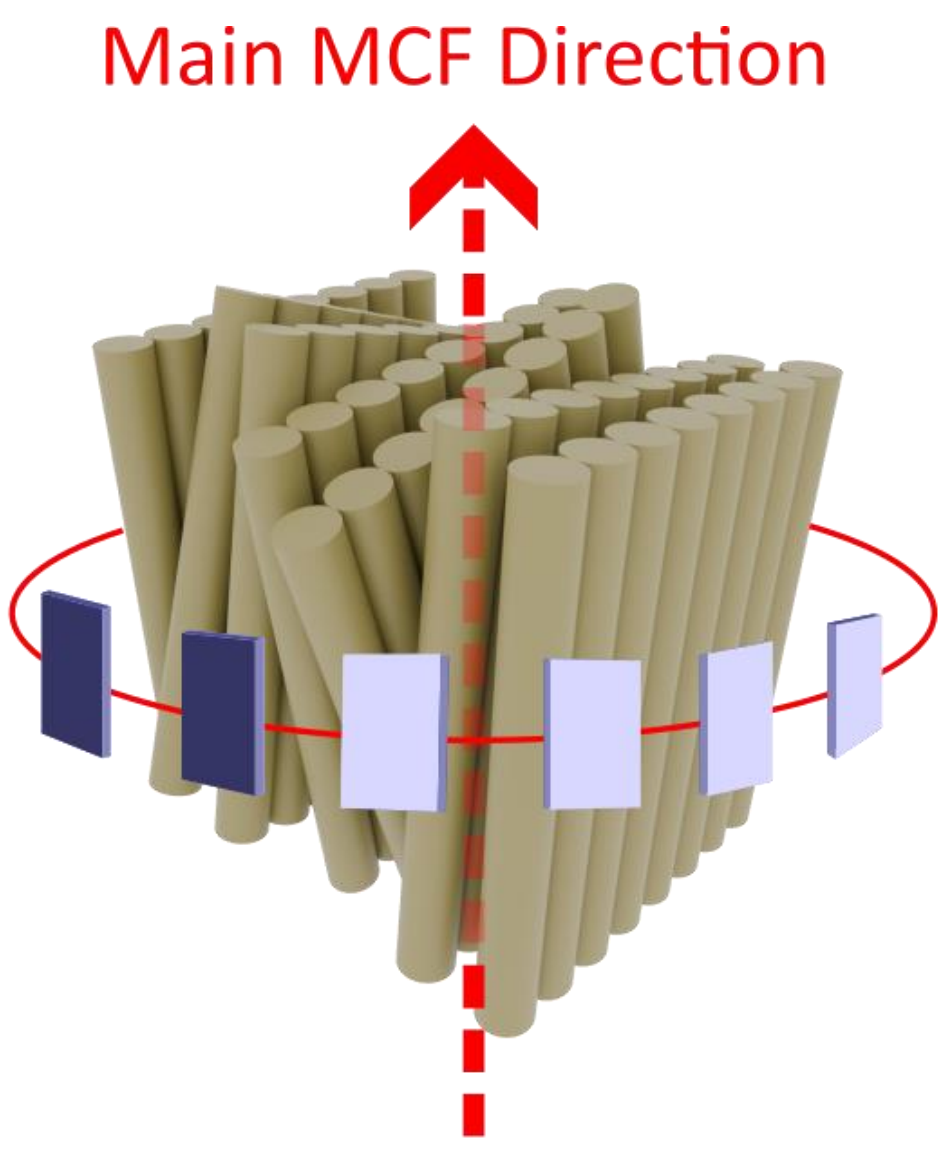

Figure S4: Schematic of subset of mineral particles oriented orthogonally to the main MCF direction in a voxel encompassing multiple lamellae.

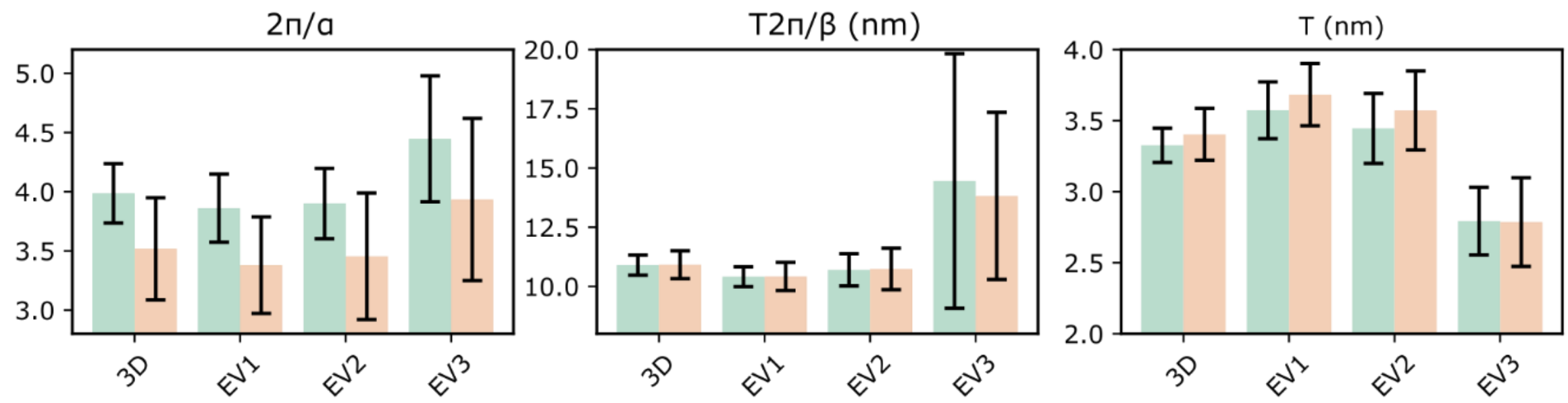

Figure S5: Mineral size and arrangement parameters calculated along the Eigenvectors of the Mineral scattering signal ($q = 0.18 - 0.5nm^{-1}$). The smallest eigenvector (EV3) indicates the direction of the weakest mineral scattering, displaying short-range order ($2\pi/\alpha$) and inter-platelet distance ($\frac{T2\pi}{\beta}$) values up to 18% and 38% higher than in the strongest scattering direction (EV1). The T-parameter is up to 25% lower in the weakest scattering directions than in the strongest. These values display the extent of the 3D directional differences in the mineral size and arrangement at the $25\mu m$length scale.

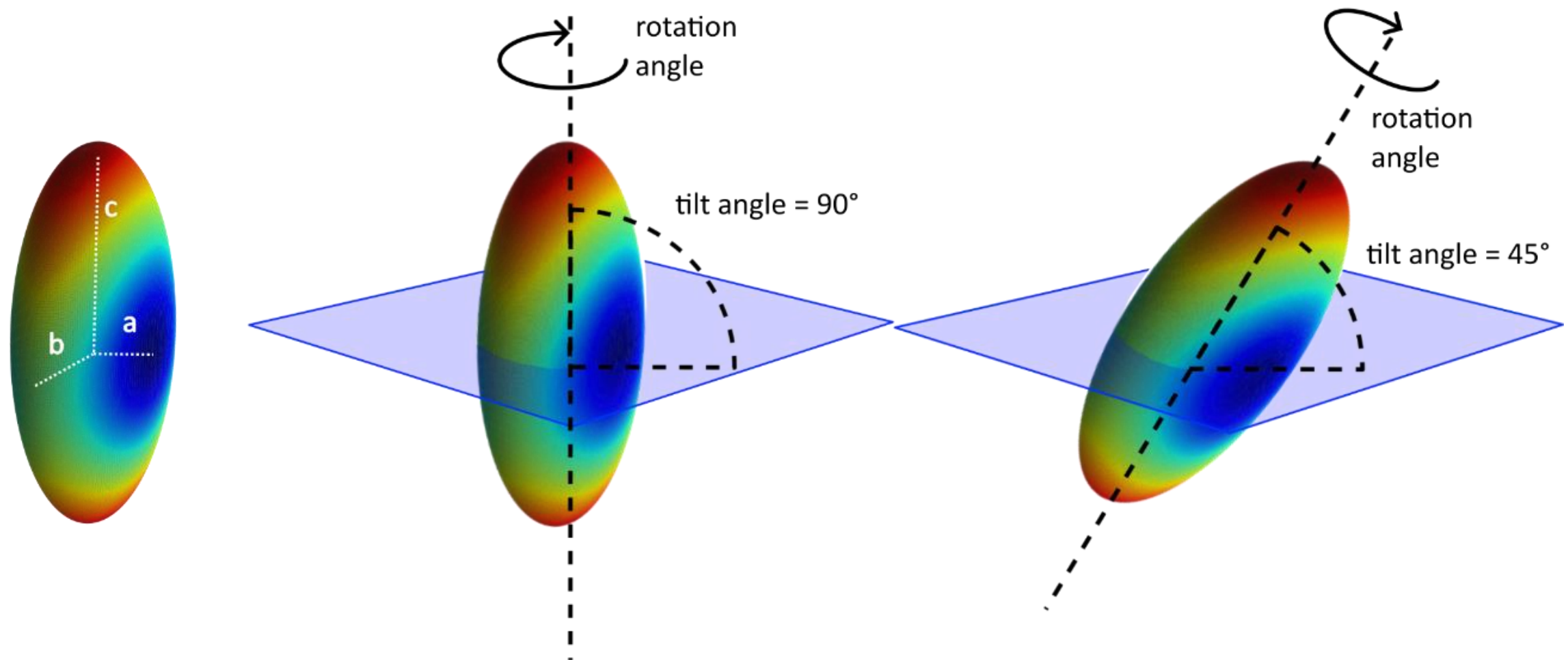

Figure S6: Representation of a generalized ellipsoid with half axes a,b,c, with the out-of-plane angle and transverse measurement plane (blue plane).

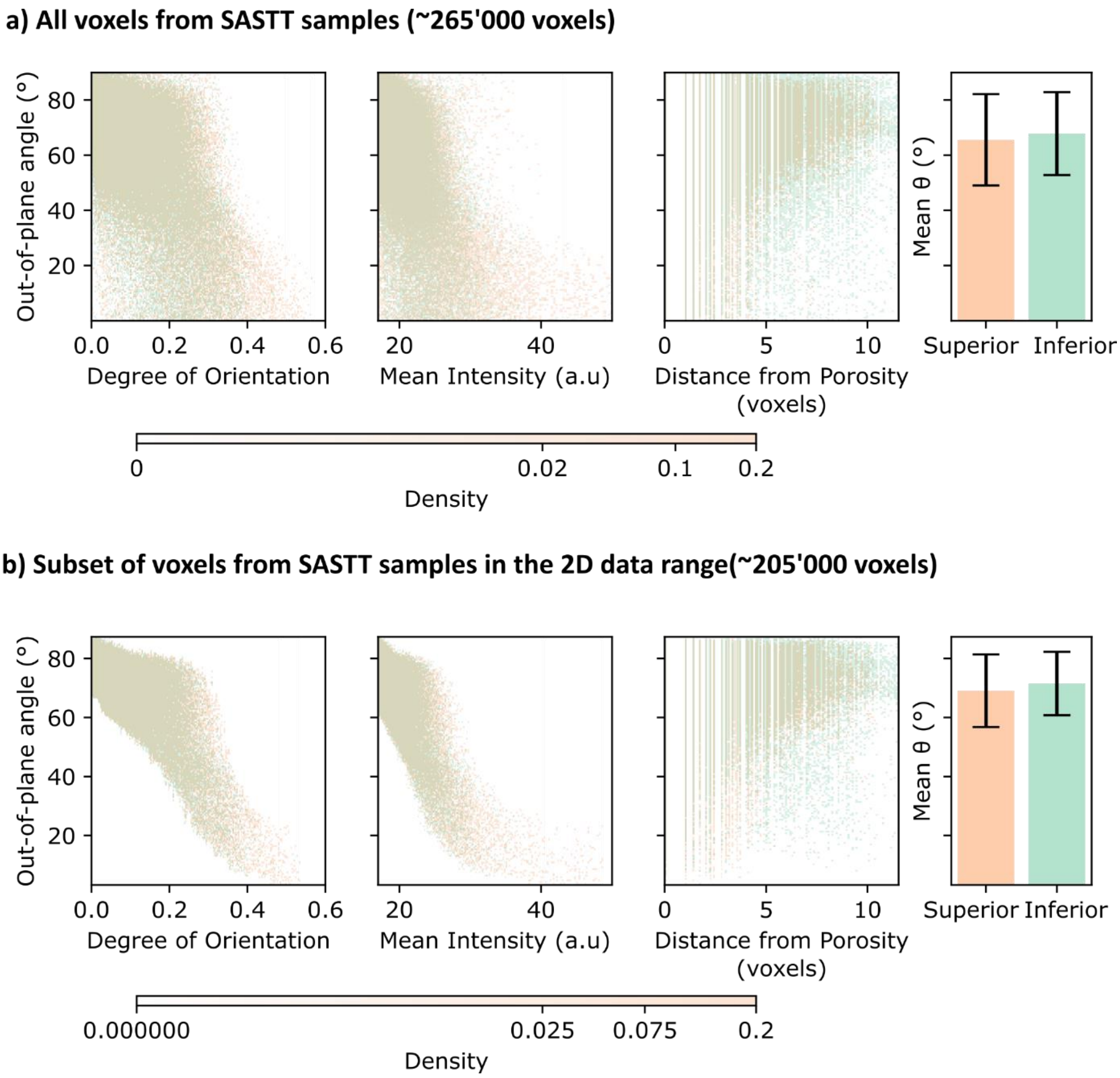

Figure S7: Correlations between out-of-plane angle of meridional collagen scattering with the model parameters, by anatomical location: Degree of orientation and mean intensity of the transverse slices, distance of the voxel from porosity and anatomical location of the voxel. (a) All voxels from the 4 SASTT samples. (b) Subset of voxels in the 2D data range (between the 5th and 95th percentiles of mean intensity for a given 2D degree of orientation, see Figure 3d). The individual correlations of the out-of-plane angle with the degree of orientation and mean intensities are broad, but can be combined to obtain a stronger correlation with the out-of-plane angle (Figure 3c)

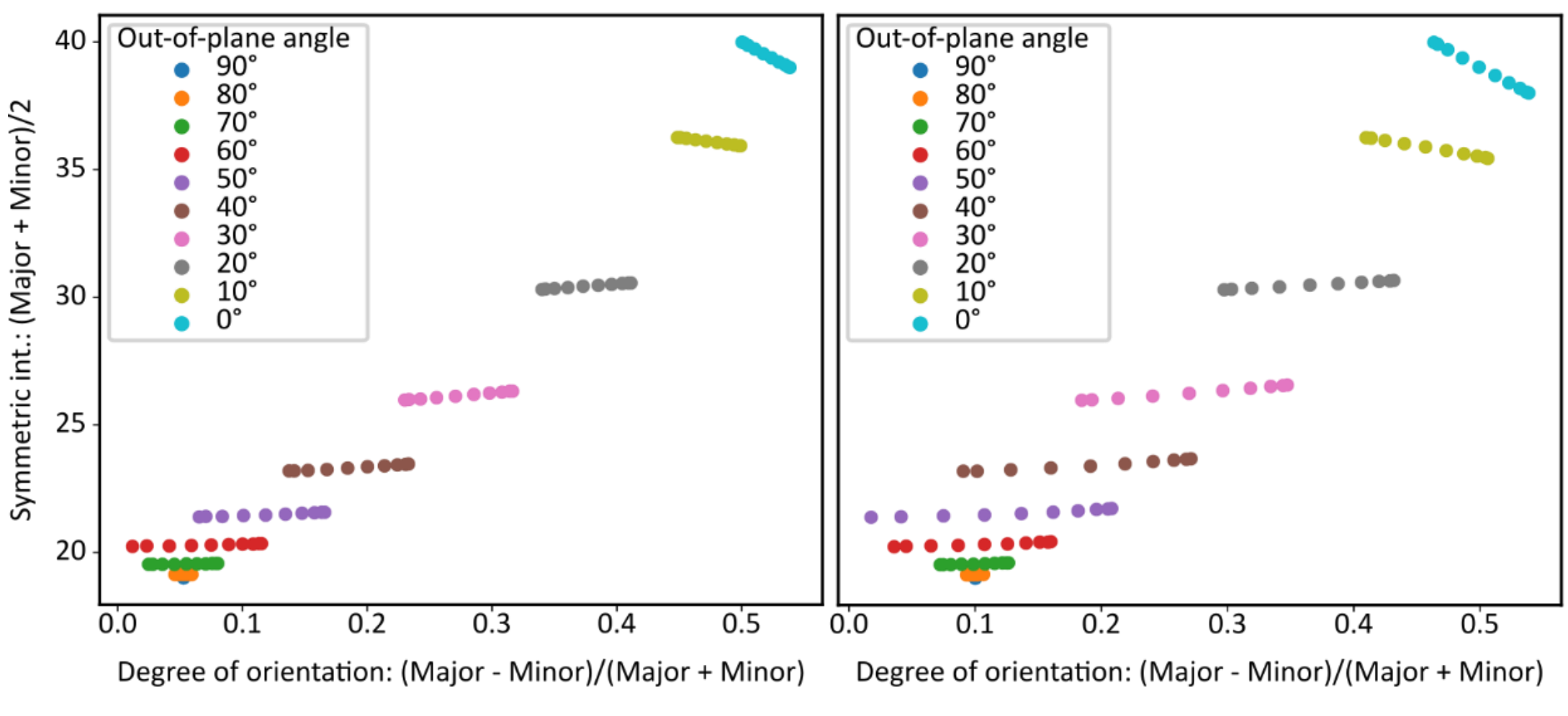

Figure S8: Scatter plot of mean intensity vs measured degree of orientation in the transverse plane for two generalized ellipsoids (Left: a = 18, b=20, c=60, FA = 0.64 ; Right; a = 18, b=20, c=60, FA = 0.66) tilted and rotated at various angles. Each colo

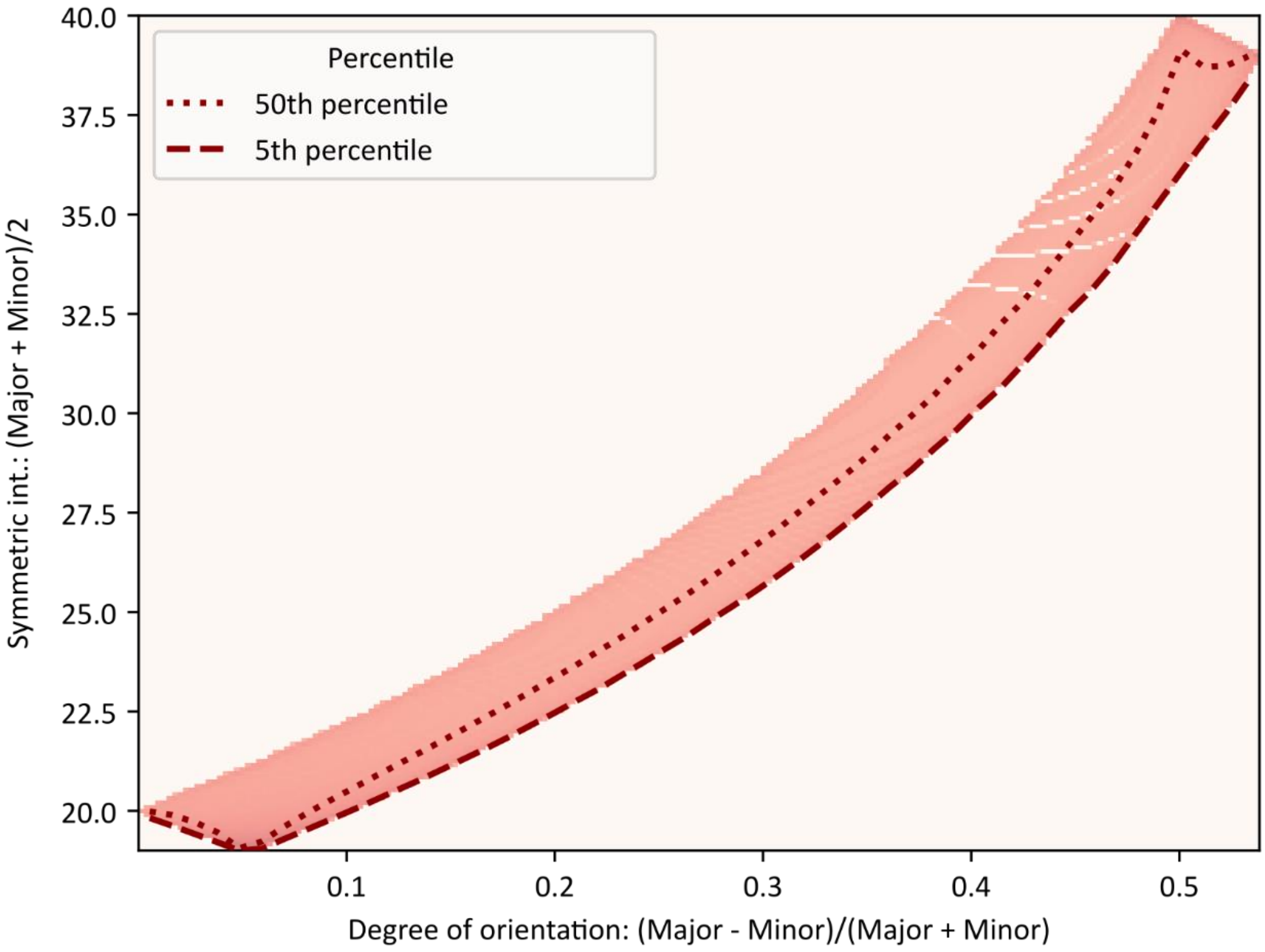

Figure S9: Density plot of mean intensity vs measured degree of orientation in the transverse plane for a generalized ellipsoid (a = 18, b=20, c=60) tilted and rotated at various angles. The 50th percentile line in each degree of orientation bin illustrates the asymmetric mean intensity distribution obtained, which correlates to the distribution found in the experimental data.

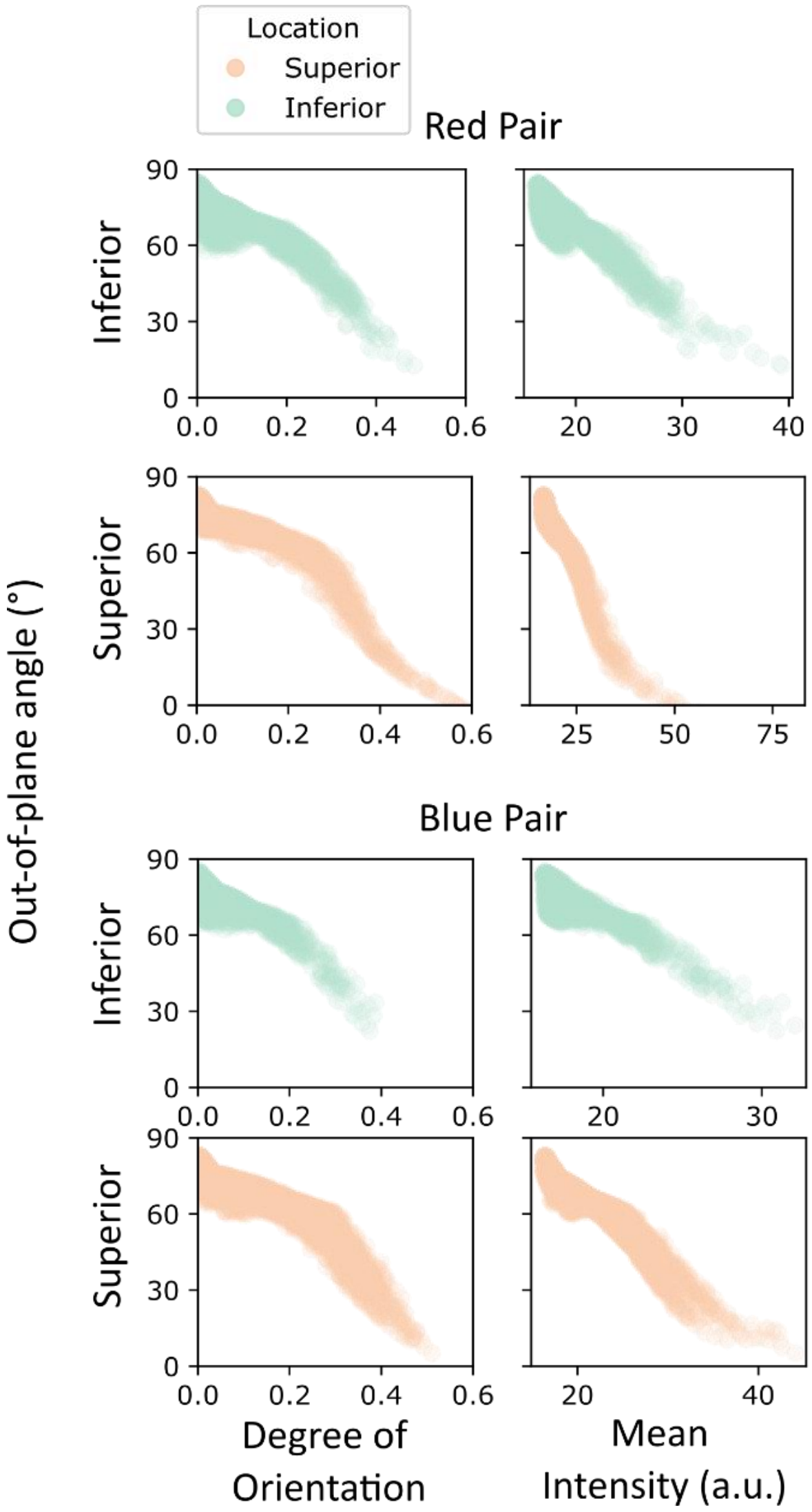

Figure S10: Pixel-by-pixel correlations between the degree of orientation and mean intensity of the meridional collagen scattering with the estimated MCF out-of-plane angle for the two sample pairs shown in Figure 4.

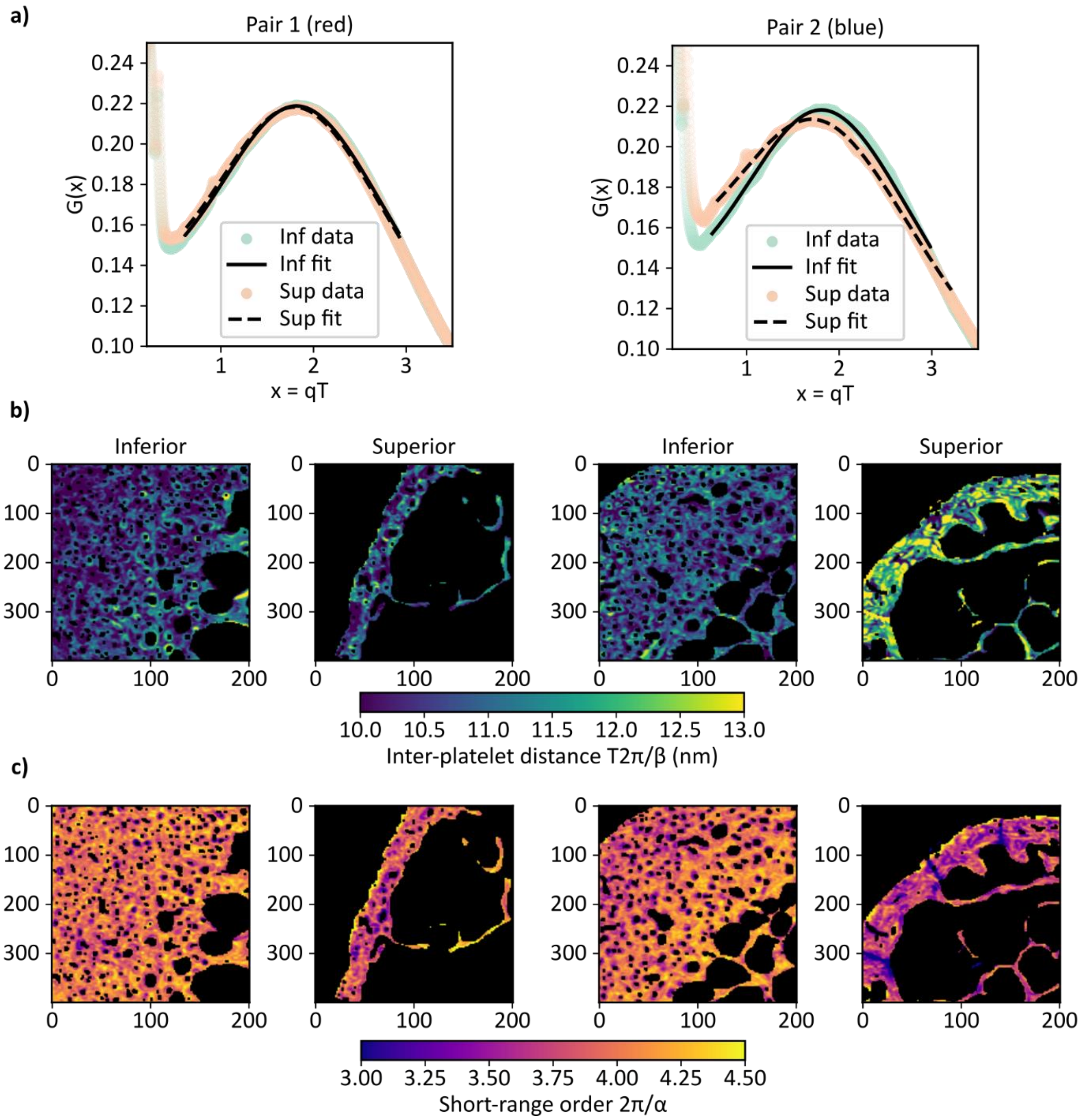

Figure S11: Mineral arrangement parameters for the example pairs shown in figure 4. a) Mean G(x) curves and the corresponding fits using the stack-of-cards model. b) interplatelet-distance parameter c) short range order parameter.

Table S2: Variance decomposition of absorption and orientation parameters in the linear mixed-effects model

	X-ray absorption	Mer. Collagen Mean Intensity	Mer. Collagen Degree of Orientation	Equ. Collagen Mean Intensity	Equ. Collagen Degree of Orientaion	Mineral Mean Intensity	Mineral Degree of Orientation	HA (002) Mean Intensity	HA (002) Degree of Orientation	MCF out-of-plane angle
**ICC_Donor (%)**	4.27	4.49	2.74	8.68	3.27	10.65	4.57	14.26	1.77	2.36
**ICC_ROI (%)**	18.30	11.63	10.31	16.82	10.97	13.29	8.48	11.66	8.40	17.35
**Residual_ Fraction (%)**	77.42	83.88	86.95	74.50	85.75	76.06	86.95	74.08	89.83	80.28

Table S3: Variance decomposition of structural parameters in the linear mixed-effects models

	T-paramenter	Mineral disorder	Inter-platelet distance	Mer. Collagen D-spacing	Equ. Collagen D-spacing	Collagen ratio (Mer. Vs Equ.)	Crystal Size	HA (002) D-spacing	Alpha	Beta
**ICC_Donor (%)**	7.31	20.06	8.18	1.07	3.46	6.40	4.50	17.11	19.90	12.85
**ICC_ROI (%)**	13.83	9.74	12.45	4.35	18.71	13.64	4.74	6.51	9.94	10.61
**Residual_ Fraction (%)**	78.86	70.20	79.37	94.59	77.83	79.95	90.76	76.38	70.16	76.54

Table S4: Results from the linear mixed-effects model for all the analyzed parameters. The coefficients and p-values are color-coded by their level of significance ($p < 0.001$, $p<0.01$, $p<0.05$, $p>0.05$). All p-values are adjusted for multiple comparisons using the Benjamini-Hochberg procedure to control the false discovery rate.

		**Age**		**Sex[M]**		**Location [Sup]**		**MCF Out-of-plane angle**		**Thickness**	
**Parameter**	**Intercept β_0**	**Coeficient β_1**	**p-value**	**Coeficient β_2**	**p-value**	**Coeficient β_3**	**p-value**	**Coeficient β_4 (per 10°)**	**p-value**	**Coeficient β_5 (per μm)**	**p-value**
X-ray absorption (cm^{-1})	9.48e+00	7.28e-03	0.914	-4.40e-02	0.953	-1.02e+00	<1e-16	2.63e-01	<1e-16	2.96e-02	<1e-16
Coll. Mer. Mean int.	2.82e+01	-7.92e-04	0.914	1.41e-02	0.953	1.94e-01	<1e-16	-1.44e+00	<1e-16	4.93e-04	<0.001
Coll. Mer. DOO	3.85e-01	-1.06e-05	0.914	2.84e-04	0.953	6.64e-03	<1e-16	-4.80e-02	<1e-16	1.29e-04	<1e-16
Coll. Equ. Mean int.	1.61e+01	-4.19e-03	0.914	6.37e-02	0.736	-5.91e-01	<1e-16	3.97e-01	<1e-16	1.28e-02	<1e-16
Coll. Equ. DOO	4.34e-02	2.86e-05	0.914	-8.11e-04	0.736	6.02e-03	<1e-16	-3.22e-03	<1e-16	9.56e-07	0.604
Mineral Mean int.	-6.68e+02	-1.92e+00	0.914	1.77e+00	0.956	-7.83e+01	<1e-16	2.09e+02	<1e-16	3.89e+00	<1e-16
Mineral DOO	6.10e-01	-2.26e-04	0.914	3.80e-03	0.872	4.91e-02	<1e-16	-6.34e-02	<1e-16	1.67e-04	<1e-16
HA (002) Mean int.	3.29e+02	4.93e-02	0.914	-4.55e+00	0.736	-5.21e-01	<0.001	-2.43e+01	<1e-16	-3.15e-01	<1e-16
HA (002) DOO	6.55e-01	2.86e-04	0.914	6.80e-03	0.736	1.99e-02	<1e-16	-7.17e-02	<1e-16	1.11e-04	0.001
MCF out-of-plane angle	72.43e+00	2.10e-02	0.914	-6.97e-01	0.736	-2.35e+00	<1e-16	N/A	N/A	5.00e-02	<1e-16
Power law exp.	1.56e+00	-4.25e-04	0.914	-1.41e-02	0.378	8.00e-02	<1e-16	1.14e-02	<1e-16	-3.83e-05	0.007
T-parameter (nm)	2.22e+00	1.54e-03	0.914	5.07e-02	0.953	1.44e-01	<1e-16	7.83e-02	<1e-16	-2.43e-04	<0.001
Crystal Size (nm)	7.54e+01	-1.49e-02	0.914	-2.48e-01	0.953	1.25e+00	<1e-16	-3.80e+00	<1e-16	1.34e-01	<1e-16
Coll. Mer. D-spacing (nm)	5.81e+01	6.85e-04	0.914	-1.80e-02	0.736	-4.25e-01	<1e-16	4.41e-01	<1e-16	6.36e-03	<1e-16
HA (002) D-spacing (nm)	3.43e-01	-1.32e-05	0.914	1.31e-04	0.736	2.49e-04	<1e-16	2.97e-04	<1e-16	1.21e-05	<1e-16
Coll. Equ. D-spacing (nm)	7.57e+01	-6.82e-03	0.914	-4.52e-01	0.953	-1.24e+00	<1e-16	1.44e+00	<1e-16	1.57e-02	<1e-16
Coll. ratio (Mer. vs Equ.)	1.65e+00	9.99e-05	0.914	-2.42e-03	0.736	4.06e-02	<1e-16	-9.72e-02	<1e-16	-6.72e-04	<1e-16
Alpha	1.56e+00	-4.12e-04	0.914	-1.66e-02	0.736	7.91e-02	<1e-16	9.14e-03	<1e-16	-2.02e-04	<1e-16
Beta	1.59e+00	-2.04e-04	0.914	-7.81e-03	0.736	1.91e-02	<1e-16	1.58e-02	<1e-16	-1.17e-04	<1e-16
Mineral Disorder	4.05e+00	8.00e-04	0.914	4.03e-02	0.087	-1.86e-01	<1e-16	-2.06e-02	<1e-16	4.90e-04	<1e-16
Inter-platelet Distance (nm)	8.97e+00	6.96e-03	0.914	2.45e-01	0.953	4.48e-01	<1e-16	-1.79e-01	<1e-16	3.53e-04	0.001

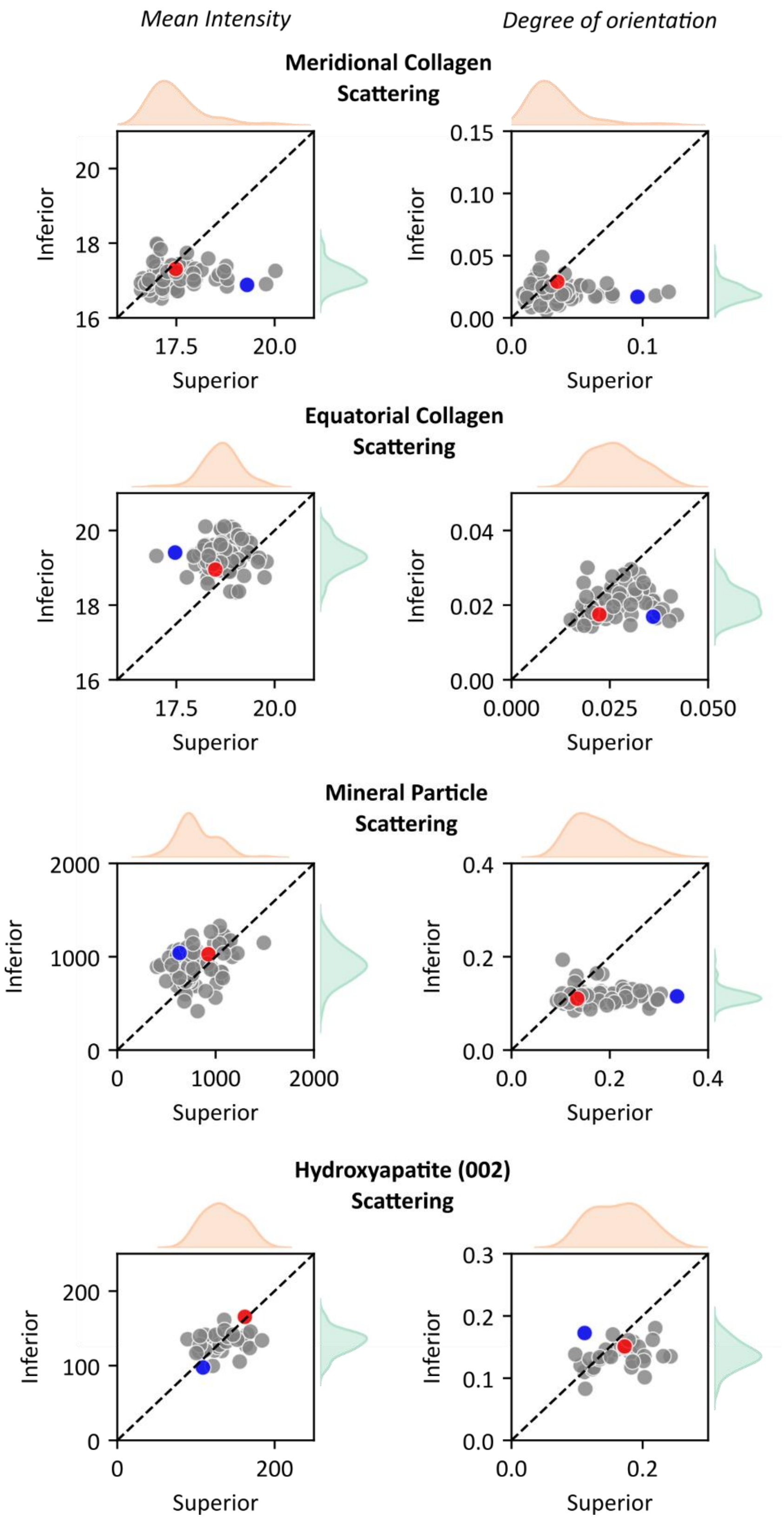

Figure S12: Mean Intensity (left column) and 2D degree of orientation (right column) of the 4 analyzed scattering signals: Meridional collagen scattering (row1), equatorial collagen scattering (row 2), mineral particle scattering (row3), hydroxyapatite 002 scattering (row 4). For each femoral neck (n = 78), a scatter point represents the median value in the superior quadrant (x-axis) and inferior quadrant (y-axis). The blue and red points indicate the sample pairs from figure 4.

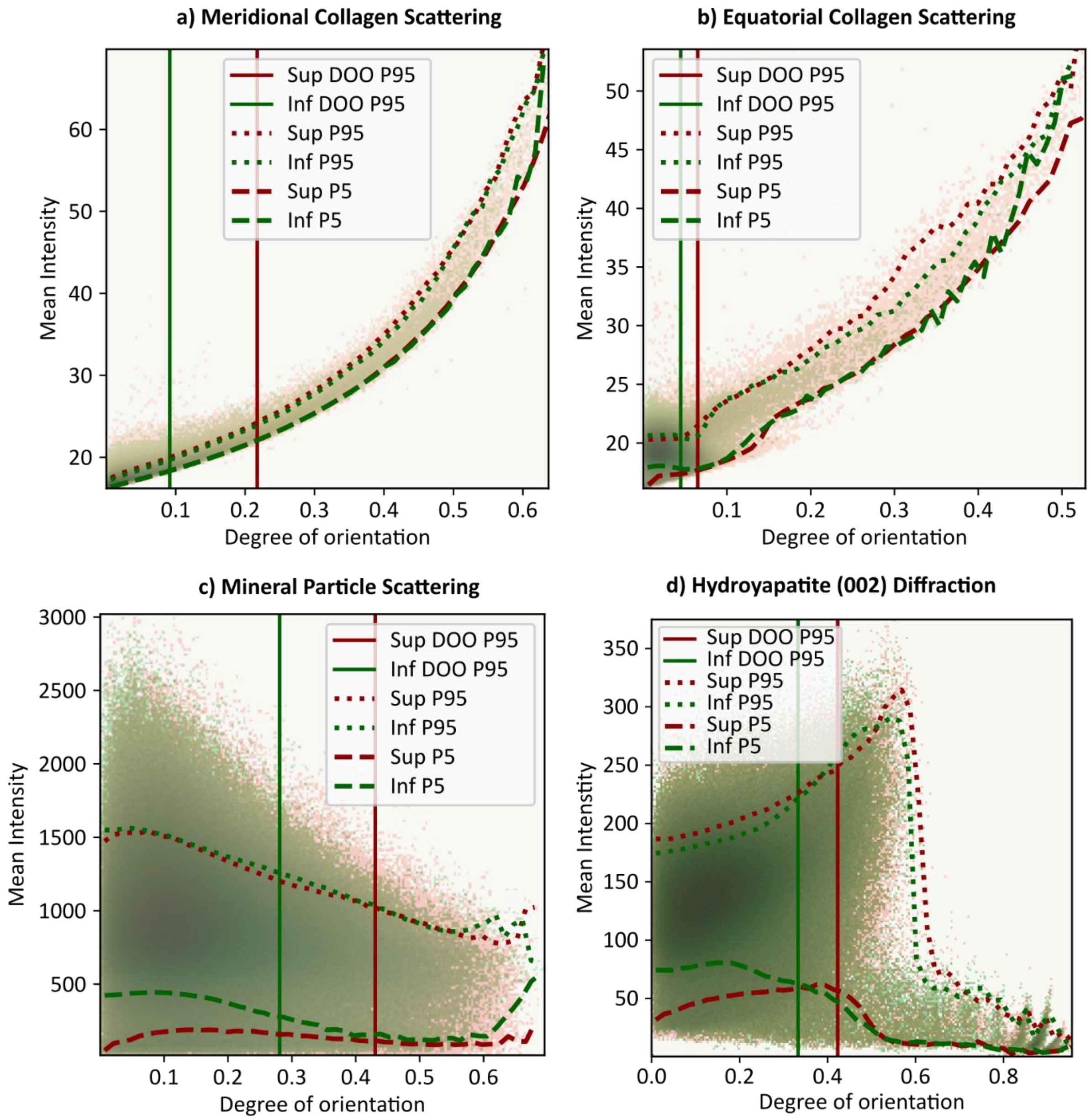

Figure S13: Correlations between degree of orientation and mean intensity for the 4 analyzed scattering signals for the 2D dataset (4.3M pixels), separated by anatomical location. The 95th percentiles in the degree of orientation distributions (DOO) and the 5th and 95th percentiles in the intensity per degree of orientation bin are shown for each anatomical location. The meridional collagen scattering (a) shows the strongest correlation, suggesting it has a high dependency on the out-of-plane angle. The greater variation in the other signals (b-d) suggest greater structural variation in the respective scatterers. All 4 signals suggest a systematic change in out-of-plane angle between the two anatomical locations, considering the shift towards higher degree of orientations (Sup and Inf DOO p95 lines). The higher mean intensity in the mineral particle scattering (c) In plot (d), the data in the bottom right is caused by scratches in the samples and was not used in the statistical analysis.

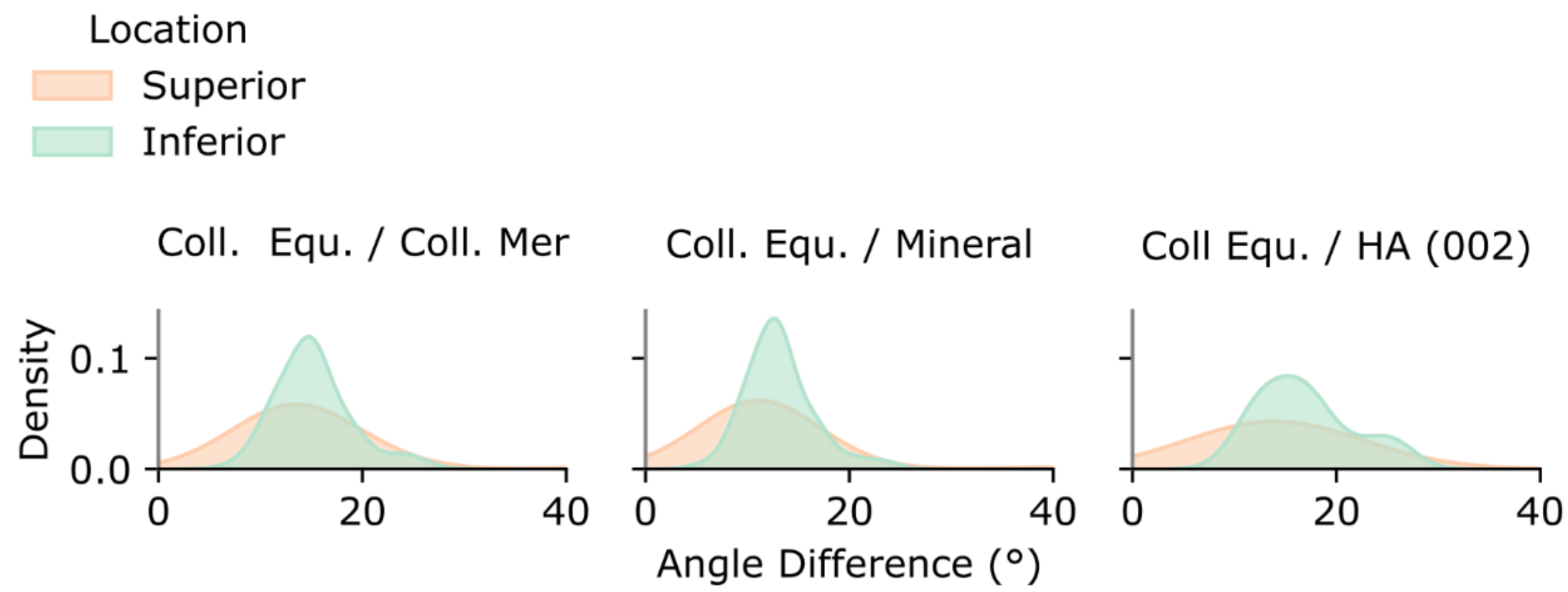

Figure S14: Orientation misalignment of the collagen equatorial scattering with the collagen meridional scattering, mineral particle and hydroyapatite (002) scattering. The misalignment is greater on the inferior side in all cases, but the distributions are broader on the superior side.

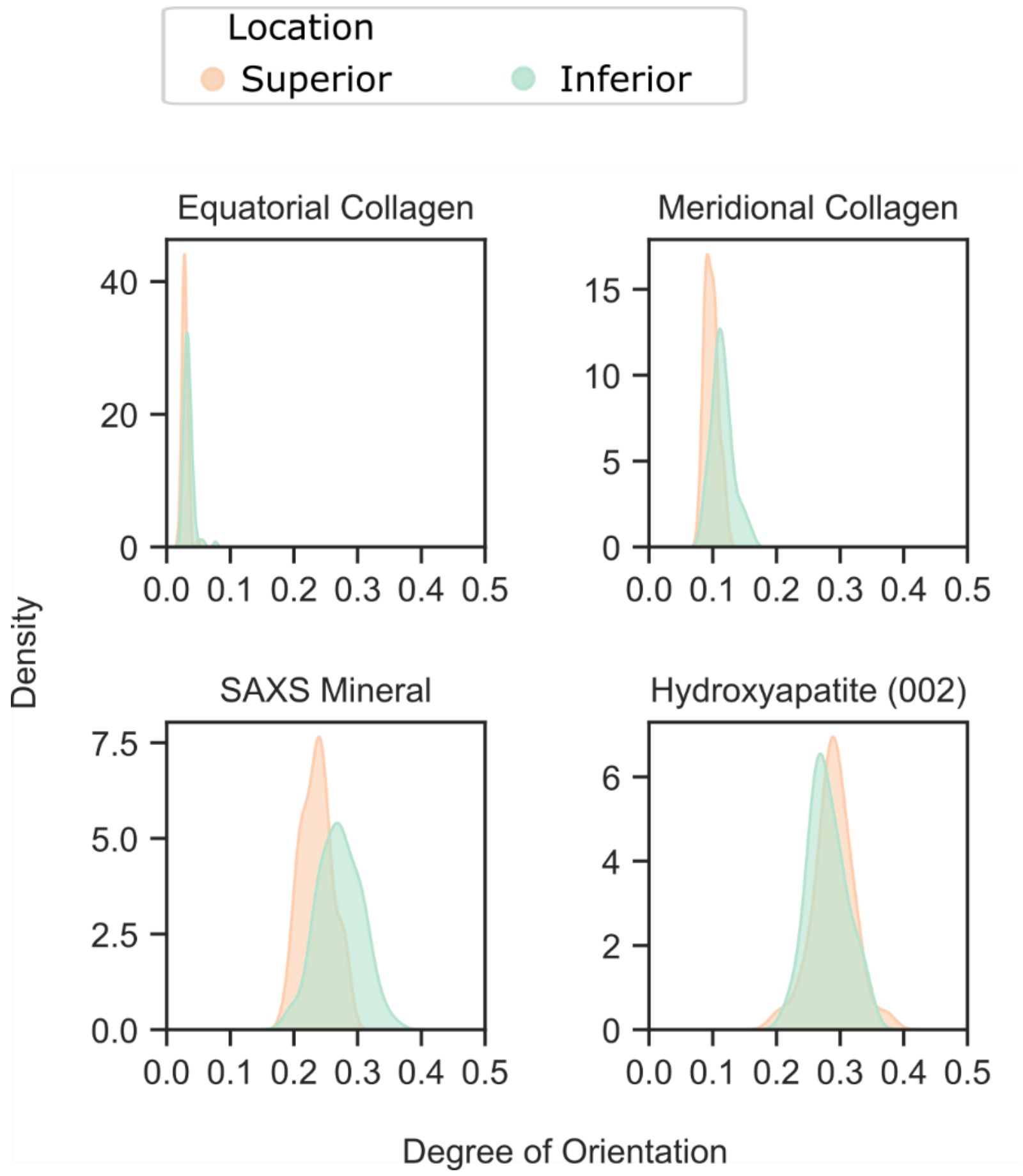

Figure S15: 2D degree of orientation of the 4 analyzed scattering signals, per anatomical location. Both collagen signals display very low degree of orientation ($\lesssim 0.15$), due to the predominantly longitudinal MCF orientation. Both mineral signals show slightly higher degree of orientation ($\sim 0.2 - 0.4$), indicating a larger amount of oriented mineral platelets and HA crystals in the transverse plane. This could indicate the presence of extrafibrillar mineral being oriented due to the osteonal growth direction. The differences between anatomical locations reflect the out-of-plane angle difference.

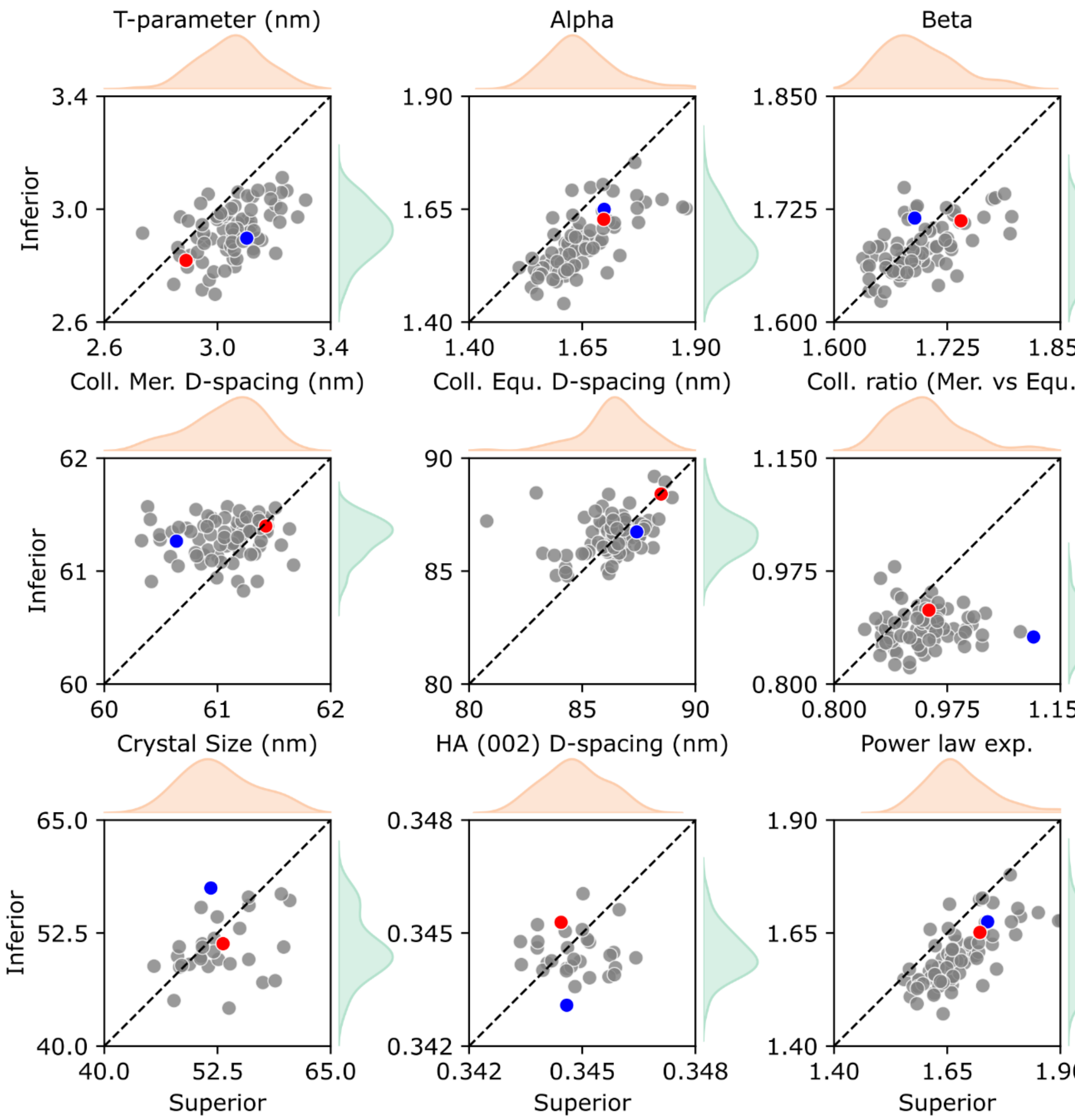

Figure S16: Structural parameters measured in the 2D scanning SAXS/WAXS dataset. For each femoral neck (n = 78), a scatter point represents the median value in the superior quadrant (x-axis) and inferior quadrant (y-axis). The blue and red points indicate the sample pairs from figure 4.

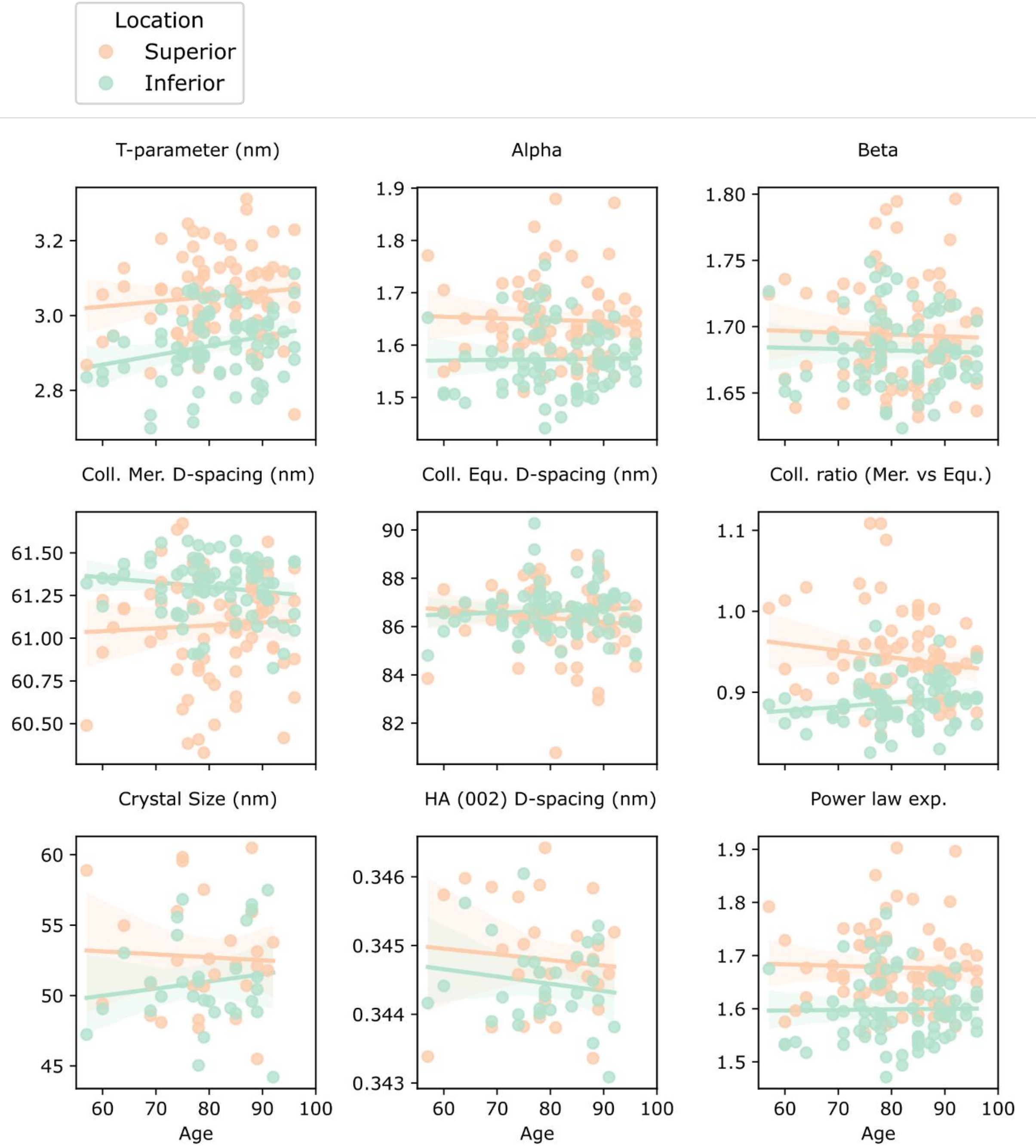

Figure S17: Correlations between structural parameters and age of donors, separated by anatomical location. No significant correlations were noted, p-values are shown in Table S4.

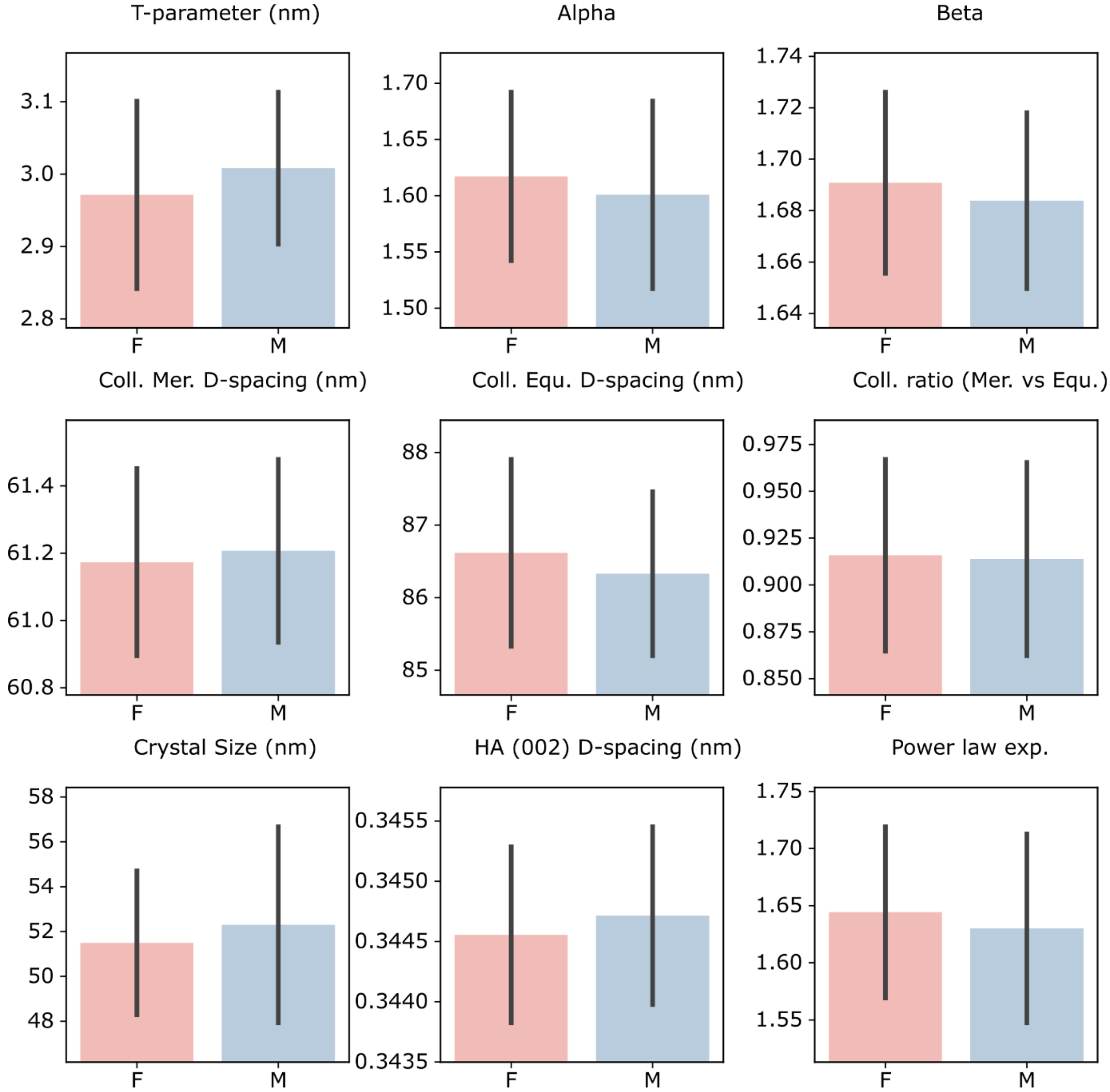

Figure S18: Correlations between structural parameters and sex of the donors. No significant correlations were noted, p-values are shown in Table S4.

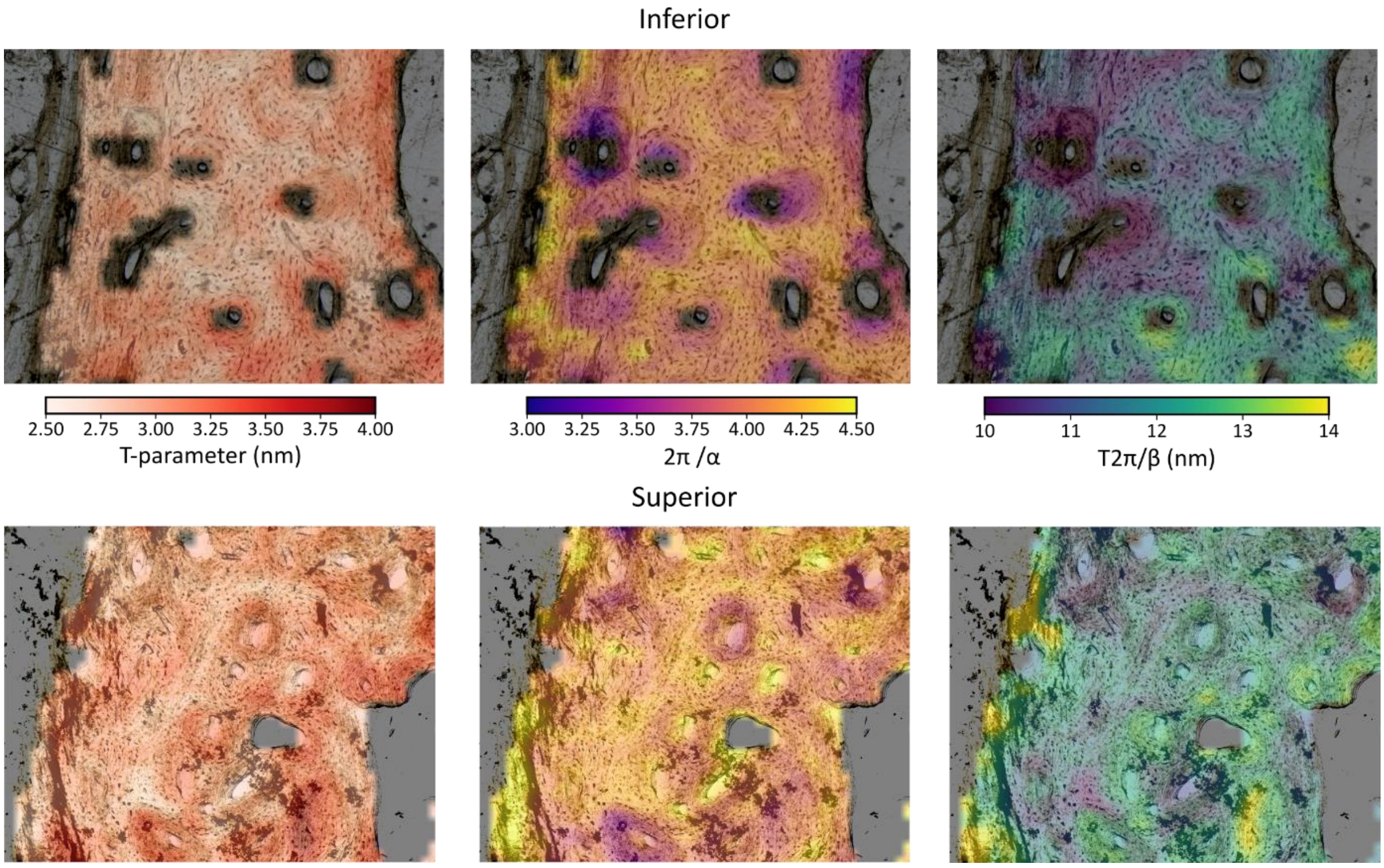

Figure S19: Mineral thickness (T-parameter) and arrangement parameters (short-range ordering $2\pi/\alpha$ and inter-platelet distance $T2\pi/\beta$) for an inferior and superior sample presenting different microstructural characteristics. The inferior side presents osteons with lower short-range order (lower $2\pi/\alpha$) and lower inter-platelet distance, while the osteons on the superior side display more variation.
